# Minocycline as adjunctive treatment for treatment-resistant depression: study protocol for a double blind, placebo-controlled, randomized trial (MINDEP2)

**DOI:** 10.1186/s12888-020-02553-9

**Published:** 2020-04-15

**Authors:** Muhammad Ishrat Husain, Clare Cullen, Madeha Umer, Andre F. Carvalho, Stefan Kloiber, Jeffrey H. Meyer, Abigail Ortiz, Yuliya Knyahnytska, M. Omair Husain, Justine Giddens, Breno S. Diniz, Wei Wang, Allan H. Young, Benoit H. Mulsant, Zafiris J. Daskalakis

**Affiliations:** 1 Centre for Addiction and Mental Health, Toronto, Ontario Canada; 2 Department of Psychiatry, University of Toronto, Toronto, Ontario Canada; 3 IMPACT Strategic Research Centre, Deakin University School of Medicine, Geelong, VIC Australia; 4 Kings College London, London, UK

**Keywords:** Depression, Minocycline, Inflammation, Anti-inflammatory, Clinical trial

## Abstract

**Background:**

Available evidence suggests that adjunctive treatment with immunomodulatory medications may be effective in the treatment of major depressive disorder (MDD). A pilot trial of the tetracycline minocycline as adjunctive treatment in treatment-resistant depression (TRD), produced promising results, however, a larger scale trial is needed to confirm the antidepressant actions of this drug.

**Methods:**

This is a 12-week double blind, placebo-controlled, randomized trial of minocycline as an add-on to standard antidepressants for adults (age > 18) with DSM-5 major depressive episode, who have failed to respond to at least two adequate trials of antidepressant treatment. It is a parallel-arm study with 50 participants in each group. The primary outcome measure is change in 17-item Hamilton Depression Rating Scale (HRSD-17) total scores from baseline to week 12. Secondary measures include the Clinical Global Impression (CGI) scale, World Health Organization Quality of Life Short Version (WHOQOL-BREF) and the Generalized Anxiety Disorder scale (GAD-7). Peripheral inflammatory biomarkers will be collected at baseline, week 6 and 12.

**Discussion:**

If minocycline is well tolerated and effective in reducing depressive symptoms in patients with TRD, it would warrant genuine consideration as a treatment option for TRD. Additionally, if results demonstrate that minocycline has antidepressant properties, and that changes in inflammatory status are associated with its antidepressant action, it will inform the development of individualized treatment for a subset of patients with MDD.

**Trial registration:**

Clinicaltrials.gov identifier: NCT03947827. Registered 13th May, 2019.

## Background

Major depressive disorder (MDD) is the leading cause of ill health and disability worldwide [1]. Over 11% of Canadians experience a major depressive episode during their lifetime treatments, a high proportion of patients neither respond nor achieves remission [2]. At least a third of all MDD patients experience treatment-resistant depression (TRD), defined as a failure to respond to two adequate medication trials [3]. Those suffering from TRD are left suffering from a significant decline in their social and occupational functioning and higher rates of all-cause mortality [4]. Persistent symptoms in TRD often translate into exponential increases in work loss and medical costs compared to more responsive forms of illness [5]. Current drug treatment strategies for TRD such as augmentation of antidepressants with atypical antipsychotics and lithium were discovered by serendipity, do not target a specific pathology, have limited efficacy and are associated with problematic adverse effects (e.g. sedation, fatigue, weight gain, diabetes, tardive dyskinesia). Therefore, evaluation of novel targets is an unmet need.

There are several lines of research indicating evidence for abnormal inflammatory processes in MDD. First, there is a substantial body of work indicating that peripheral markers of inflammation are abnormally elevated, in MDD, with abnormal profiles of circulating pro- and anti-inflammatory biomarkers [6]. Moreover, longitudinal studies report that high plasma pro-inflammatory cytokine levels precede, and thus potentially predispose to depressive symptoms [7, 8]. Evidence also suggests that peripheral inflammation is associated with a more severe course of illness [9], and more prominent in people who are resistant to antidepressants [10, 11]. Second, administration of pro-inflammatory stimuli like lipopolysaccharide is associated with depressive behaviors in humans and animals [12, 13]. Third, all four positron emission tomography studies of translocator protein (TSPO) binding in currently depressed participants with early onset MDD, report elevated levels in the regions sampled, including the prefrontal and anterior cingulate cortex. Elevated levels of TSPO are associated with gliosis in neuropsychiatric diseases [14–17]. Gliosis involves a transformation of microglia and astroglia from surveillance functions to inflammatory and/or repair roles, to respond to various types of brain injury [18] changing morphology from branched to globular, with a large increase in cell volume, while migrating to and engulfing the site of insult.

The second-generation tetracycline antibiotic minocycline has known immunomodulatory effects and is commonly used for the treatment of inflammatory skin conditions such as acne. It is also being investigated in CNS diseases associated with inflammation such as multiple sclerosis, stroke and Parkinson’s disease [19]. It has a long half-life of 12–18 h and is the most lipid-soluble of the tetracycline antibiotics, allowing for good penetration into the cerebrospinal fluid (CSF) and CNS through the blood-brain barrier [20]. It is usually well tolerated and has low propensity to produce antibiotic resistance [20]. Although minocycline has multiple actions that make it a candidate treatment for MDD, many of these actions are mediated through its immunomodulatory effects. These include suppressing signaling within microglia including mitogen-activated protein kinases, and major histocompatibility complex II expression, as well as release of cytokines such as TNF-α, and IL-6 [21]. Minocycline also suppresses TSPO expression in rodents exposed to immunogenic stimuli. Minocycline has a number of additional actions, exhibited largely in cell lines and animal models, including reduction of oxidative stress and apoptosis; and modulation of glutamate and monoamine neurotransmission. Additionally, minocycline has been shown to normalize glucocorticoid levels through its actions on the hypothalamic pituitary adrenal (HPA) axis [22]. Another inflammatory pathway that has been implicated in the pathophysiology of MDD is the kynurenine pathway, which can influence production of quinolinic acid, affecting glutamate neurotransmission and synthesis of serotonin. Minocycline acts on this pathway and has been shown to reduce activity of the enzyme indoleamine 2,3-dioxygenase (IDO), thereby diverting tryptophan metabolism towards serotonin synthesis and away from production of quinolinic acid [23].

We led the first pilot randomized controlled trial (RCT) (*n* = 41) of the tetracycline antibiotic minocycline as adjunctive treatment in TRD demonstrating that it led to a significant reduction in depressive symptoms as measured by the 17-item Hamilton Rating Scale for Depression (HRSD-17) [24] compared to placebo. We found a large effect size for minocyline (− 1.21, Cohen’s d = 0.98) [25], the magnitude of which deserves further investigation. Moreover, minocycline was well tolerated, as there was no significant difference in frequency of adverse effects between groups. Though these findings are encouraging, a more definitive trial with a larger sample size is required to confirm the antidepressant actions of minocycline.

### Aims

The current study aims is to examine whether minocycline added to standard antidepressants for 3 months in patients with TRD leads to a reduction in depressive symptoms as measured by the 17-item Hamilton Rating Scale for Depression (HRSD-17) compared with placebo added to antidepressants. In addition, we will assess the tolerability of minocycline added to antidepressants, compared to placebo added to antidepressants. We expect that minocycline will significantly reduce the severity of depressive symptoms compared to the placebo group. We also predict that there will be no significant difference in the frequency of serious adverse effects between both groups.

As an exploratory aim, we will investigate whether TRD patients with evidence of abnormal inflammatory processes at baseline, are more likely to respond to minocycline, and whether changes in pro- and anti-inflammatory cytokines and C-reactive Protein (CRP) and natural log CRP (lnCRP) predict a response to minocycline. We hypothesize that higher baseline plasma pro-inflammatory cytokines, CRP and lnCRP will predict response to minocycline. Furthermore, we predict that changes in plasma pro-inflammatory cytokine levels, CRP and lnCRP will mediate treatment response to minocycline.

## Methods

This study is a 12 week, double blind, placebo-controlled, randomized trial of minocycline added to standard oral antidepressants for patients suffering from a Diagnostic and Statistical Manual of Mental Disorders, 5th Edition (DSM-5) [26] major depressive episode, who have failed to respond to adequate dose-duration trials of at least two antidepressants. It will be a two parallel-arm study with 50 participants in each arm, giving a total of 100 participants. The study is currently in the recruitment stage.

### Participants

#### Recruitment

Participants will be recruited from the outpatient program of the General Adult Psychiatry and Health Systems division at the Centre for Addiction and Mental Health (CAMH) in Toronto, Ontario, Canada through physician referrals, reviewing new referrals to the clinic for potential eligibility and recruitment posters.

The research analyst (RA) will provide an oral and written description of the study to the interested patient explaining what the study involves, possible risks and potential benefits. They will be informed that their participation is voluntary and that they have the right to withdraw from the study at any time. The RA must obtain signed informed consent from participants before beginning any study related activities.

#### Eligibility

Patients are selected for study enrolment based on the inclusion and exclusion criteria listed in Table 1. During the study, investigators can decide to withdraw a participant for urgent medical reasons, or if the situation of a participant changes such that he or she is no longer eligible to participate.

**Table 1:** Inclusion and exclusion criteria

Subject Eligibility	
**Inclusion Criteria**	1) are voluntary outpatients who are competent to consent to treatment;
	2) have a DSM–5 diagnosis of non-psychotic MDD, single or recurrent, based on the Structured Clinical Interview for DSM-5 (SCID-5) (R. L. Spitzer et al., 2015);
	3) are male or female between the ages of 18–80;
	4) total score of > 3 on the Antidepressant Treatment History Form (ATHF) (M. A. Oquendo et al., 1999; Maria A. Oquendo et al., 2003)
	5) have a baseline Hamilton Rating Scale for Depression (HRSD-17) > 14;
	6) if a woman of child-bearing potential, are on a medically acceptable form of birth control such as oral contraceptives, contraceptive injections (Depo-Provera), intrauterine devices (IUD), contraceptive patch, male partner sterilization, abstinence, or barrier methods (condom or diaphragm) plus spermicide,
	7) are currently taking one of the following antidepressants: Escitalopram, Citalopram, Sertraline, Venlafaxine, Duloxetine, Mirtazapine or Bupropion
**Exclusion Criteria**	1) meet DSM-5 substance use disorder criteria within the past 3 months;
	2) have a concomitant major unstable medical illness;
	3) are pregnant or intend to get pregnant during the study;
	4) have a SCID-5 diagnosis of any psychotic disorder, bipolar disorder, obsessive compulsive disorder, or post-traumatic stress disorder (current or within the last year). While patients with psychotic depression subtype have been shown to respond and remit with minocycline treatment, we exclude these patients in order to pursue a conservative study with a homogeneous non-psychotic TRD sample first and will use such information to guide future research development with patients with psychotic features;
	5) Have a DSM-5 diagnosis of borderline personality disorder as assessed by a study investigator;
	6) have possible or probable dementia (based on the Informant Questionnaire on Cognitive Decline in the Elderly (Jorm & Jacomb, 1989) that will be administered to any subject with a baseline score of < 26 on the Montreal Cognitive Assessment (MoCA) (Nasreddine et al., 2005);
	7) prior history of intolerance to any of the tetracyclines or presence of any contraindication to tetracyclines;
	8) abnormal readings in hematology (Hemoglobin, White Blood Cell count, platelet count), liver functions (ALT, AST, bilirubin) or renal function test (BUN, creatinine);
	9) patients with Myasthenia Gravis;
	10) concomitant treatment with anticoagulants, diuretics, retinoids, ergot alkaloids, antacids containing aluminium, calcium or magnesium, bismuth and zinc salts, quinapril

This study will be conducted in accordance with the principles of the Declaration of Helsinki and has received approval from the Research Ethics Board (REB) at CAMH (REB# 135/2018) and is registered on ClinicalTrials.gov (Identifier: NCT03947827). The independently chaired Trial Steering Committee (TSC) will meet twice in the first year of the study and annually thereafter. The TSC will monitor overall trial progress, conduct and will also advise on scientific credibility of the trial throughout its various stages. In addition, the independent Data Safety Monitoring Board (DSMB) will meet annually and will be the only body with access to the un-blinded data.

### Intervention

Participants will be randomized to receive either minocycline 200 mg daily or placebo added to one of the following standard oral antidepressants: escitalopram, citalopram, sertraline, venlafaxine, duloxetine, mirtazapine or bupropion. Participants will not be permitted to switch antidepressants during the trial. Should participants consider a medication change prior to randomization, they will be switched to one of the pre-specified antidepressants and will be re-screened for eligibility after 6 weeks. Participants will be permitted to take benzodiazepine medications and/or acetaminophen or acetylsalicylic acid (Aspirin) when required. Participants will not be permitted to take any other augmentation treatment for major depressive disorder such as, atypical antipsychotics (e.g. quetiapine, aripiprazole, olanzapine, risperidone, lurasidone, brexipiprazole), lithium and lamotrigine, or any regular anti-inflammatory medication.

Participants will not be permitted to start a psychosocial intervention or psychotherapy during the study period, however those who were already engaged in these treatments at the screening stage will be permitted to continue their treatment.

#### Randomization and blinding

Participants will be randomly allocated to a treatment group using a random permuted block method, prepared by the study statistician. On notification of a new participant by the RA, the trial pharmacist will assign that participant to either group according to the block and drugs will be prepared accordingly. All capsules, whether active drug or placebo, will be in identical blister packs. Patients, referring clinicians, the research team and RA’s conducting study assessments will remain concealed from the allocation for the duration of the study. The pharmacy will maintain a drug allocation list and emergency un-blinding slips for each participant should there be a need for emergency un-blinding. To assess the integrity of blinding procedures, upon participant completion of the study participants and independent raters will be asked to complete a conventional guess form asking whether they believe participants received minocycline or placebo as a treatment.

#### Clinical procedures

The CAMH research pharmacy will dispense medications at baseline, week 2 and week 6. Minocycline will start at an oral dose of 100 mg daily and will be increased after 1 week to 100 mg twice daily. This dose has been shown by substantial literature to produce consistent anti-inflammatory effects in inflammatory disorders. It was also the dose used in our pilot trial [27]. Treatment compliance will be assessed by the pharmacy at each dispensing visit using pill counts.

### Outcomes

#### Clinical screening assessments

Eligible participants will undergo clinical bloodwork (Hemoglobin, White Blood Cell count, platelet count, ALT, AST, bilirubin, BUN, creatinine) and will be screened with the Structured Clinical Interview for DSM-5 (SCID-5) and HRSD-17 to determine study eligibility. The SCID-5 assesses DSM-5 psychiatric diagnoses and will be used to confirm psychiatric inclusion and exclusion criteria.

**Fig. 1:**
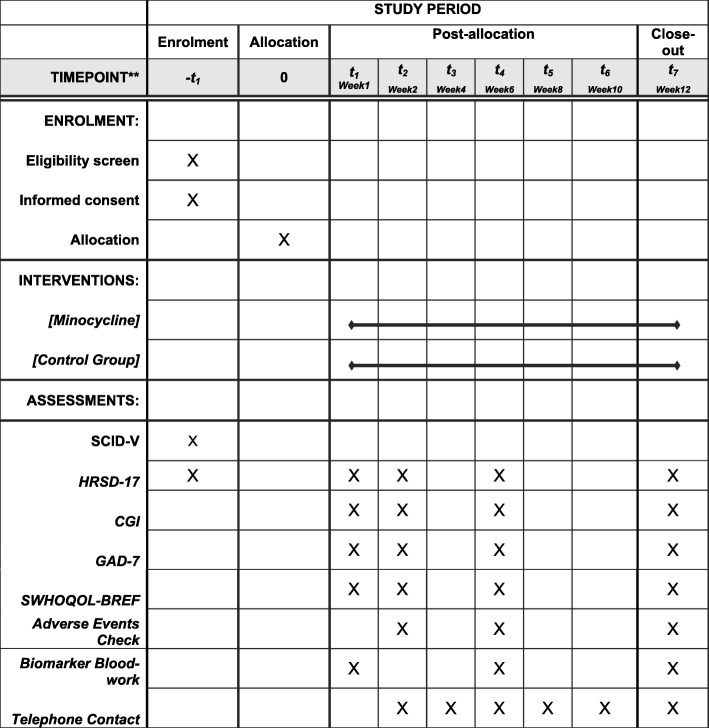
SPIRIT Flow Diagram

The principal investigator will review all results from the screening visit before making a determination of eligibility for each participant. If deemed eligible to continue, participants will attend four assessment visits (baseline, weeks 2, 6 and 12) in addition to fortnightly telephone check-ins from the RA. Table 2 and Fig. 1 detail the assessment schedule.

**Table 2:** Assessment Schedule

Assessment	Screening	Baseline	Week 2	Week 4	Week 6	Week 8	Week 10	Week 12	Ad hoc
Medical Confirmation of Eligibility, SCID-5	x								
Clinical Bloodwork	x								
Randomization	x	x							
HRSD-17	x	x	x		x			x	
CGI		x	x		x			x	
GAD-7		x	x		x			x	
WHOQOL-BREF		x	x		x			x	
Minocycline dispensed		x	x		x				
TAU/Concomitant medication		x	x		x			x	
Adverse Event Checks		x	x		x			x	x
Physical health measures		x							
Biomarkers (CRP, cytokines)		x						x	
Telephone contact				x		x	x	x	x

#### Measures

The primary outcome will be changes in depressive symptoms from baseline to week 12 on the 17-item Hamilton Rating Scale for Depression (HRSD-17). Secondary outcomes include response defined as > 50% reduction of the HRSD score and remission defined as a score of < 8 on the HRSD-17. Other secondary clinical outcome measures will include: changes in the Clinical Global Impression (CGI) scale [28], a measure of illness severity; World Health Organization Quality of Life Short Version (WHOQOL-BREF) [29], a self-report questionnaire assessing four domains of quality of life; and the Generalized Anxiety Disorder scale (GAD-7) [30], a self-report measure of severity for generalized anxiety disorder. Adverse effects will be monitored using a rating scale that has been specifically designed for minocycline. This rating scale has been used in previous studies [27].

#### Biomarkers

Participants will be asked to provide blood samples at baseline, weeks 6 and 12. Blood will be collected using EDTA tubes by antecubital venipuncture in the morning, after overnight fasting. The blood will be processed according to standard laboratory procedures for plasma separation. Plasma aliquots will be stored in a − 80 °C freezer until the biomarkers’ analyses. The biomarkers will include CRP, inflammatory cytokines (CCL-2, IL-6, IL-8, IL-12, IL-23, TNF-alpha, and IL-1). The selection of cytokines is based on meta-analyses indicating that these proteins are elevated in patients with MDD, compared to healthy controls, and are therefore putative biomarkers of abnormal inflammatory processes in MDD [31]. We will use commercially available multiplex Luminex assays for the cytokine analyses.

### Sample size and power

A sample of 100 subjects, 50 per group, results in 80% power to detect a standardized effect size Cohen’s f = 0.13 [32], which is a medium effect size (equivalent to Cohen’s d = 0.3). A recent network meta-analysis of RCTs of antidepressants vs. placebo indicates that available antidepressants have a pooled effect size of 0.3 [33] and thus an effect size of 0.3 or above would indicate clinical utility of minocycline. The calculation assumes 20% dropout rate, two tailed tests and significance level of 0.05. The power calculation was conducted using G*Power 3.1.9.2 [34] and assuming a repeated measure ANOVA model where the interaction between treatment group and time is the target.

### Statistical analysis

Initial descriptive analysis will present the profile of the subjects and investigate group differences at baseline on main demographics and clinical measures. Group comparison will be conducted through Chi-square tests for categorical variables and non-parametric Mann Whitney U test for continuous variables. If significant differences between groups are found the variable may be added to the models testing the relevant study hypotheses.

For the primary hypothesis, the change in HRSD-17 scores from baseline to week 12 will be compared between groups. A mixed effect model that includes treatment group, time and their interaction as fixed effects, and individual subjects as random effects will be adjusted to the data. A linear contrast will be used to test the difference between groups in changes from baseline to week 12 in HRSD scores. Two tailed tests and significance level of 0.05 will be used. To compare response and remission rates between groups logistic regression will be used. To compare frequency of adverse events, generalized linear mixed model will be used, with Poisson or negative binomial distribution, where time and treatment group and their interaction are fixed effects with subjects as random effects.

The exploratory hypothesis that baseline pro-inflammatory cytokines/CRP levels will affect response to minocycline will be tested by adding the baseline pro-inflammatory cytokines/CRP in the final model used to test our primary hypothesis, and interacting it with the already existing interaction between treatment group and time, therefore resulting in a three way interaction. The pro-inflammatory cytokines/CRP will be considered moderators if the three-way interaction is significant considering significance level of 0.05. In that case, exploratory plots that look at the group effect at different levels of the moderators will be used to study the nature of the moderation.

In order to test the hypothesis that changes in peripheral levels of pro-inflammatory cytokines/CRP will be a mediator in the causal path between treatment and change in HRSD scores, an initial descriptive analysis will be conducted to look at the bivariate association between treatment group and change in pro-inflammatory cytokine/CRP levels, and between change in pro-inflammatory cytokine/CRP and change in HRSD scores. A final model that specifies the change in pro-inflammatory cytokine/CRP and in HRSD as random growth slopes (Latent Growth Model) will be adjusted to the data, where the pro-inflammatory cytokine/CRP slope is specified as causing the HRSD slope. The treatment group is then specified as causing both slopes. The mediation effect is tested by the significance of the indirect effect from treatment group to change in HRSD that goes through change in pro-inflammatory cytokine/CRP. This model will be adjusted in Mplus 8.4 [35] and the indirect effect is tested through using bootstrap resampling.

Intention-to-treat analysis will be conducted where subjects are assigned to groups as randomized. Mixed effect models, adjusted through maximum likelihood can use all available information in the data avoiding removal of dropout subjects from the analysis [36]. Diagnostic analysis will be conducted through checking residuals for outliers, influential data points and normality. If outliers or influential points are found, a sensitivity analysis will conducted after removing such points. Subgroup analysis will be carried out with respect to age and gender by adding a treatment with covariate interaction into the primary analysis model.

## Discussion

Approximately one third of patients with MDD have TRD [37], leading to poorer clinical, functional and social outcomes. Although the mechanisms of treatment resistance are not yet fully understood, TRD patients represent a significant portion of all patients with MDD, making the need to understand its pathophysiology and find alternative treatments an important research goal.

To our knowledge, this is only the second RCT of minocycline for the treatment of TRD. It is intended to be a larger scale study than the previous pilot trial [27], led by the same principal investigator and with more than double the number of participants (*n* = 41 vs. *n* = 100). If this study leads to similar findings as the pilot trial, i.e. that adjunctive minocycline is efficacious in reducing depressive symptoms, it has great potential to translate in to clinical practice. Minocycline is an inexpensive, easily available antibiotic drug and could be an affordable and accessible treatment option for TRD patients.

Given that we cannot yet predict the likelihood of an individual patient’s response to a particular antidepressant, this study presents a unique opportunity to identify markers of inflammatory pathology in MDD while also assessing if treatment with minocycline can change such markers at clinically relevant doses. If the biomarker analysis demonstrates that minocycline has antidepressant properties, and that changes in inflammatory processes are associated with its antidepressant action, it will enhance the understanding of the pathophysiological mechanisms involved in MDD and its treatment. Furthermore, should the study provide data that identifies abnormal inflammatory processes as treatment targets in depression, it could lead to the development of ‘precision medicine’ using immunomodulatory therapeutics for a sub-group of patients that have treatment-resistant symptoms as a result of underlying abnormalities in the inflammatory response system.

## Data Availability

Not applicable.

## Abbreviations

CAMH: Centre for Addiction and Mental Health
CGI: Clinical Global Impression
CNS: Central nervous system
CRP: C- reactive Protein
CSF: Cerebrospinal fluid
DSMB: Data Safety Monitoring Board
DSM-5: Diagnostic and Statistical Manual of Mental Disorders, 5th Edition
Diagnostic and HPA: GAD-7: Generalized Anxiety Disorder scale; Hypothalamic Pituitary Adrenal axis
HRSD: Hamilton Rating Scale for Depression
MDD: Major depressive disorder
PET: Positron-emission tomography
RCT: Randomized controlled trial
REB: Research Ethics Board
SCID-V: Structured Clinical Interview for DSM-5
TNF: Tumor Necrosis Factor
TRD: Treatment resistant depression
TSC: Trial Steering Committee
WHOQOL-Bref: World Health Organization Quality of Life Short Version
